# Solution‐crystallization and related phenomena in 9,9‐dialkyl‐fluorene polymers. II. Influence of side‐chain structure

**DOI:** 10.1002/polb.23797

**Published:** 2015-08-17

**Authors:** Aleksandr Perevedentsev, Paul N. Stavrinou, Paul Smith, Donal D. C. Bradley

**Affiliations:** ^1^ Department of Physics and Centre for Plastic ElectronicsImperial College LondonLondonSW7 2AZUnited Kingdom; ^2^ Department of MaterialsEidgenössische Technische Hochschule (ETH) ZürichVladimir‐Prelog‐Weg 58093ZürichSwitzerland

**Keywords:** conjugated polymers, crystallization, microstructure, polyfluorene, spectroscopy

## Abstract

Solution‐crystallization is studied for two polyfluorene polymers possessing different side‐chain structures. Thermal analysis and temperature‐dependent optical spectroscopy are used to clarify the nature of the crystallization process, while X‐ray diffraction and scanning electron microscopy reveal important differences in the resulting microstructures. It is shown that the planar‐zigzag chain conformation termed the β‐phase, which is observed for certain linear‐side‐chain polyfluorenes, is necessary for the formation of so‐called polymer‐solvent compounds for these polymers. Introduction of alternating fluorene repeat units with branched side‐chains prevents formation of the β‐phase conformation and results in non‐solvated, i.e. melt‐crystallization‐type, polymer crystals. Unlike non‐solvated polymer crystals, for which the chain conformation is stabilized by its incorporation into a crystalline lattice, the β‐phase conformation is stabilized by complexation with solvent molecules and, therefore, its formation does not require specific inter‐chain interactions. The presented results clarify the fundamental differences between the β‐phase and other conformational/crystalline forms of polyfluorenes. © 2015 The Authors. Journal of Polymer Science Part B: Polymer Physics published by Wiley Periodicals, Inc. J. Polym. Sci., Part B: Polym. Phys. **2015**, *53*, 1492–1506

## INTRODUCTION

 The possibility to process conjugated polymers from solution is one of the key drivers that has stimulated research on printable/plastic electronics.^1^, ^2^, ^3^ This arises through the corresponding opportunity for cost‐effective large‐area processing, for example using gravure^4^, ^5^, ^6^, ^7^ and other high‐throughput printing modalities. However, the phase behavior of polymer solutions can be extremely complex, with factors such as solvent quality, polymer concentration, and processing temperature strongly affecting polymer chain conformation and the propensity for structure formation in solution, both of which can ultimately influence the resulting solid‐state properties following solvent removal.^8^, ^9^, ^10^, ^11^, ^12^, ^13^ Solution processability of conjugated polymers is typically enabled by the attachment of solubilizing side‐chains to the conjugated backbone;^14^, ^15^, ^16^ the alternative precursor‐route approaches^17^, ^18^, ^19^, ^20^ are little used any more. The chemical structure of the side‐chains is an additional variable that dramatically influences the polymer's overall solubility as well as the range of possible chain conformations and packing geometries.

All of these considerations have been evidenced in the synthesis and characterization of dialkyl‐substituted polyfluorenes (PFs)—a family of polymers closely related to the generally‐insoluble poly(*para*‐phenylene)s (PPPs) but which features a bridging carbon atom in the C‐9 position between alternating pairs of phenylene rings.^15^, ^21^ In addition to their outstanding optoelectronic properties,^9^, ^22^, ^23^, ^24^, ^25^, ^26^ PFs have proven to be an excellent material system for studying the interplay between various aspects of microstructure and photophysical properties.^26^, ^27^, ^28^, ^29^, ^30^, ^31^, ^32^, ^33^, ^34^ While a diverse range of side‐chain substituents has been reported,^35^, ^36^ most of the studies have focused on dialkyl‐substituted PFs, which exhibit solution‐ and solid‐state microstructures that can include several thermotropic liquid‐crystalline forms, as well as the distinct and widely‐studied β‐phase conformation.^9^, ^27^, ^31^, ^32^, ^37^, ^38^, ^39^, ^40^, ^41^, ^42^, ^43^, ^44^

In some cases, the distinction between crystalline structures formed in solution and solid‐state can become blurred since, for example, gels of these polymers may contain crystalline forms identical to those that can also be obtained by melt‐crystallization, e.g. in the case of syndiotactic polystyrene (sPS).^45^ On the other hand, crystallization is a kinetically‐controlled process and, hence, the solvent can play an important role in mediating inter‐chain interactions. In special cases, the solvent itself can co‐crystallize with the polymer, leading to the formation of composite *solvated* polymer crystals, commonly referred to as “polymer‐solvent compounds”.^45^, ^46^

**Figure 1:**
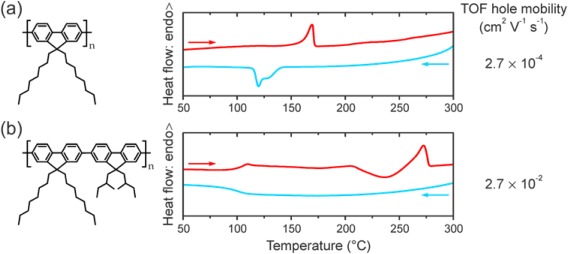
Chemical structure and first‐cooling/second‐heating DSC thermograms (blue and red lines respectively) for neat, as‐synthesized (a) PFO and (b) P(F8:F1/4). Time of flight (TOF) hole mobilities measured for “glassy” films spin‐coated from room‐temperature toluene solutions are also listed; data from Ref. 26.

In *Part I* of this study we have demonstrated that poly(9,9‐dioctylfluorene) [PFO; see Fig. 1(a)] can form a polymer‐solvent compound with organic solvents such as dodecane and 1,2,4‐trichlorobenzene. The common structural element in the reported PFO‐solvent compounds is the existence of crystalline domains comprising polymer chain segments in the β‐phase conformation. The term “β‐phase” refers to a planar‐zigzag chain conformation (inter‐monomer torsion angle ϕ = 180°) featuring a co‐planar orientation of the phenylene rings along the backbone with the octyl side‐chains located on alternate sides for adjacent fluorene repeat units. We have shown in *Part I* that the β‐phase conformation of PFO creates on‐chain cavities that allow the ordered inclusion of solvent molecules, thereby enabling the formation of a PFO–solvent compound.

However, several important questions regarding the mechanism of PFO solution‐crystallization remain open. First, it has not been established conclusively that only the β‐phase conformation, rather than any other structural feature, enables the formation of PFO‐solvent compounds. Second, the effect of inter‐chain interactions on β‐phase formation remains unclear, tending in the extreme to the potentially fundamental question: Does β‐phase formation *result from* or *cause* chain aggregation?

Many previous studies have reported that inducing the formation of β‐phase chain segments in solutions or thin films of PFO correlates with chain aggregation phenomena.^9^, ^41^, ^44^, ^47^, ^48^ Conversely, β‐phase chain segments have also been reported to be generated in cooling/warming cycles for PFO films on quartz substrates—a process speculated to rely on thermal stresses^28^—and in solutions cast on a water surface and subsequently transferred to a substrate via the Langmuir‐Blodgett process.^49^ The existence of a causal relationship between β‐phase chain segment formation and aggregation has not been explored in much detail. In one study, Bright et al.^50^ used temperature‐dependent optical spectroscopy to investigate the formation of the β‐phase in di‐*n*‐alkyl‐substituted PFs (side‐chain length *R* = 6–10 carbon atoms) in methylcyclohexane (MCH) solutions. They observed the spectroscopic signatures of β‐phase formation upon cooling PF7, PF8 (i.e. PFO) and PF9 (that is, *R* = 7, 8, and 9 carbons atoms, respectively) solutions and attributed the effect to van der Waals interactions between alkyl side‐chains providing sufficient energy to overcome the steric energy barrier to planarization of the backbone. They concluded, thereby, that β‐phase chain segments form as a *result* of inter‐chain interactions. The fact that β‐phase chain segments appear even in ultradilute solutions (∼10 ng mL^−1^) where inter‐chain aggregation is extremely unlikely, was ascribed to chain folding allowing van der Waals interactions between alkyl substituents located on different segments of the same chain. In another study, Da Como et al.^51^ investigated β‐phase formation in thin films comprising an ultradilute fraction of PFO dispersed in an inert polymer matrix. Exposing these films to solvent vapor led to the formation of β‐phase chain segments that were characterized by low‐temperature polarized single‐molecule fluorescence spectroscopy. It was reported that the β‐phase segments preferentially formed in extended chains *without* spatial rearrangement of the PFO backbone, implying that inter‐chain interactions are *not* required. Earlier photoluminescence (PL) studies of PFO dispersed at 1% by weight in a polystyrene matrix had already concluded that the β‐phase spectroscopic features did not have an interchain origin.^27^

With this background in mind, we set out to compare solution‐crystallization in PFO and the alternating copolymer poly(9,9‐dioctylfluorene‐*alt*‐9,9‐di(2‐methyl)butylfluorene) [see Fig. 1(b), hereafter abbreviated as P(F8:F1/4) and previously as S50F8:50F5^26^], which comprises fluorene units alternately 9,9‐disubstituted with long (*R* = 8) linear and short (*R* = 5) branched alkyl side‐chains. We investigate the influence of intra‐chain (change in chain conformation) and inter‐chain (aggregation/crystallization) structure formation and examine the relative differences in the kinetics of the two processes, thus clarifying the principal distinction between the β‐phase and other crystalline/conformational forms in relation to polymer‐solvent compound formation.

## EXPERIMENTAL

### Materials

Poly(9,9‐dioctylfluorene) (PFO) and poly(9,9‐dioctylfluorene‐*alt*‐9,9‐di(2‐methyl)butylfluorene) (P(F8:F1/4)) were synthesized using the Suzuki route by the Sumitomo Chemical Company Ltd; see Ref. 26 for details. Both polymers underwent extensive purification before shipment and were used as received. The weight‐average molecular weights (*M*_w_) and the corresponding polydispersity indices (PDI), as determined by polystyrene‐equivalent gel‐permeation chromatography, were: *M*_w_ = 2.87 × 10^5^ g mol^−1^ with PDI = 3.0 for PFO, and *M*_w_ = 3.16 × 10^5^ g mol^−1^ with PDI = 2.7 for P(F8:F1/4). Decahydronaphthalene (“decalin”) (98%, mixture of *cis* and *trans* isomers, Acros Organics), *n*‐dodecane (99%, Acros Organics) and *n*‐hexadecane (99%, ABCR) were used as received.

### Optical Characterization of Solutions and Gels

The chain overlap concentration c* for PFO in solution with a good solvent was estimated using:^52^

(1) $$c^{*}\approx \frac{M_{n}^{\prime }}{N_{A}(\frac{4\pi }{3})R_{g}^{3}}$$

where *N*_A_ is Avogadro's number and *M*_n_′ is the number‐average molecular weight reduced by a factor of 2.7 to correct for the overestimation of the absolute molecular weight by polystyrene‐equivalent GPC as a consequence of the higher relative chain stiffness for PFO.^9^ The radius of gyration, *R*_g_, assuming a wormlike chain structure,^9^ was calculated using the Kratky–Porod equation:^53^

(2) $$R_{g}^{2}\approx \frac{l_{0}l_{p}M_{n}^{\prime }}{3M_{u}}$$

where *l*_0_ and *l*_p_ are the repeat unit and chain persistence lengths, respectively (*l*_0_ = 0.84 nm^9^ and *l*_p_ = 9.8 nm^47^), and *M*_u_ is the molar mass of the F8 repeat unit. This yielded an estimate of c* = 0.4 wt % for the PFO used in this study; the same c* value was assumed for P(F8:F1/4) due to the absence of corresponding detailed structural information.

Dilute, isolated‐polymer‐chain solutions (*c*_p_ ≪ c*) were prepared at a polymer weight fraction *c*_p_ = 0.01 wt % in decalin. Stirring at 70 °C ensured full dissolution. Gels were prepared from semidilute decalin solutions (*c*_p_ = 0.5 wt % ≈ c*) by allowing them to stand, undisturbed, at 5 °C. Absorption spectra were recorded with a dual‐beam Shimadzu UV‐2600 spectrophotometer equipped with a diffuse reflectivity (integrating sphere) attachment. Photoluminescence (PL) spectra were measured using a Jobin Yvon Horiba Fluoromax‐3 spectrofluorometer operating in a 90° optical geometry with excitation wavelength *λ*_ex_ = 390 nm. PL quantum yields of the dilute solutions were measured with the same spectrofluorometer equipped with an integrating sphere attachment. PL self‐absorption was corrected for using the procedure described in Refs. 54 and 55. For these measurements, dilute solutions were placed inside 1 mm path length quartz cuvettes (Hellma), while gels were sealed between two quartz coverslips using ∼100 µm spacers. All measurements were carried out at room temperature.

### Thermal Analysis

Polymer solutions/gels for thermal analysis and X‐ray diffraction were prepared directly in the standard low‐pressure aluminum differential scanning calorimetry (DSC) crucibles. After the addition of the required amount of solvent, the crucibles were sealed and carefully weighed before and after measurements to ensure that no solvent loss had occurred. DSC was carried out using a Mettler Toledo DSC 822e instrument that was routinely calibrated using indium standards. As the first step, all mixtures were annealed at temperatures near the boiling point of the solvent for ≥20 min to ensure that homogeneous solutions were obtained. Standard 5 °C min^−1^ heating/cooling rates were used, except for the preparation of the so‐called “slowly‐crystallized” gel samples, intended to possess the maximal degrees of crystallinity, for which the solutions were cooled at 1 °C min^−1^ and subsequently annealed at the corresponding crystallization temperature for 45 min. DSC on neat polymer samples was carried out under nitrogen flow using 10 °C min^−1^ rates.

### Critical‐Point Drying of Gels

Dried gels for X‐ray diffraction measurements were prepared from the slowly‐crystallized gels by supercritical solvent extraction using a CO_2_ critical‐point dryer (SPI Supplies). This technique allows for interface‐free removal of the solvent and minimizes the possibility of associated structural changes to the polymer due to the absence of surface tension.^56^, ^57^ The sealed DSC crucibles containing the gel samples were opened and immediately flushed with liquid CO_2_ at ∼15 °C; the samples were then left for 2 h allowing solvent exchange to take place. The temperature was then increased to 37 °C (below the *T*_g_ of both neat PFO and P(F8:F1/4)) to enable supercritical extraction of CO_2_. Due to the limited miscibility of CO_2_ with the solvents used in this study, the drying process was repeated three times. To ensure better data comparability, the as‐prepared and dried gel samples for X‐ray diffraction measurements were prepared using identical starting polymer concentration. Aerogels for scanning electron microscopy were prepared from 0.5 wt % solutions in decalin using the same critical‐point drying procedure.

### X‐Ray Diffraction

Wide‐angle X‐ray diffraction (WAXD) was performed on an Oxford Instruments XCalibur PX diffractometer using Mo‐Kα radiation (0.71 Å wavelength). Sample preparation and experimental procedure were described in detail in *Part I* of this study. PFO‐dodecane gels were prepared with concentration, expressed as polymer repeat unit molar fraction, *x*_u_ = 0.48, i.e. slightly below the compound concentration *x*_u_* = 0.5, thus avoiding complications from solvent‐deficient solution‐crystallization. P(F8:F1/4)‐dodecane gels were prepared with *x*_u_ = 0.33. Measurements were carried out with the samples cooled to −100 °C, which is below both *T*_m_ of the free solvent and the *expected* (see *Part I* for details) *T*_g_ of the polymer‐solvent compound. The two‐dimensional diffraction patterns were radially integrated following correction for background signal.

### Scanning Electron Microscopy

Aerogel samples were mounted on carbon tape and sputter‐coated with a thin platinum layer. Scanning electron microscopy (SEM) was performed with a LEO 1530 Gemini instrument using secondary electron detection.

### Temperature‐Dependent Optical Spectroscopy

**Figure 2:**
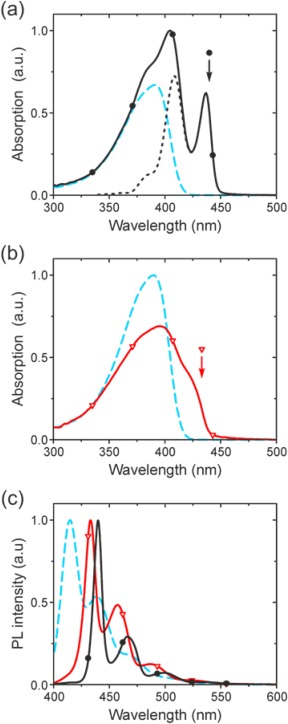
Absorption spectra of *gels* in decalin containing 0.5 wt % (a) PFO (black line with •) and (b) P(F8:F1/4) (red line with 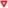). Also shown are the corresponding spectra for dilute (0.01 wt % polymer) *solutions* in decalin (dashed blue lines). Spectra are normalized at their short‐wavelength absorption tails. Also shown in (a) is the difference spectrum (dotted black line) assigned to absorption by β‐phase chain segments. The arrows in (a) and (b) indicate the spectral positions of site‐selective photoexcitation (440 and 433 nm, respectively) used for the measurements presented in Figures 6 and 7 (*vide infra*). (c) Peak‐normalized PL spectra of the gels and solutions (same lines/symbols as above).

**Figure 3:**
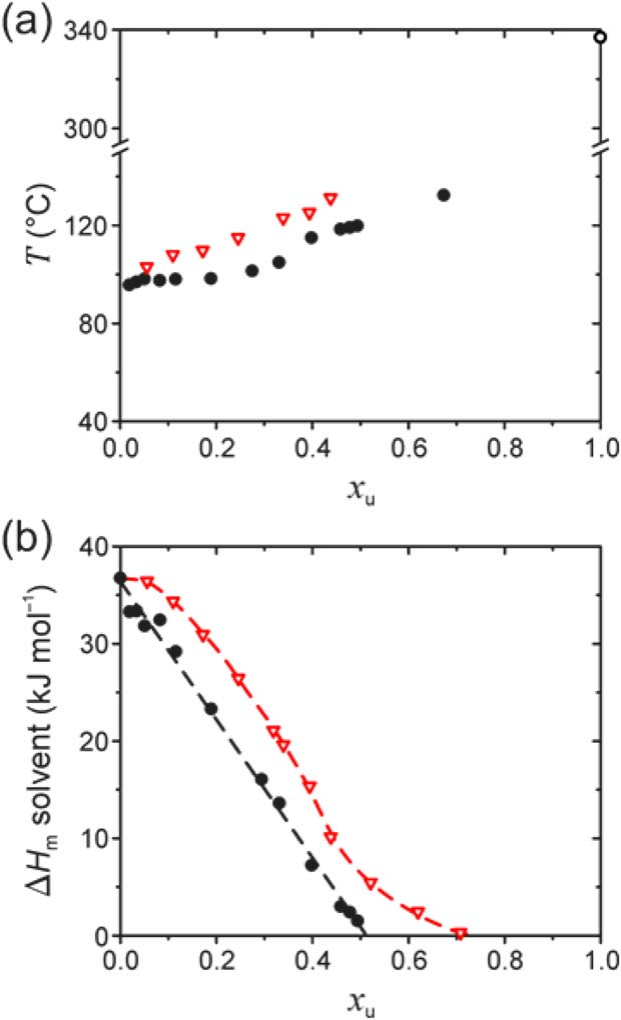
(a) Gel melting/dissolution temperature, *T*, and (b) melting/dissolution enthalpy of the solvent, Δ*H*_m_, as a function of the polymer repeat unit molar fraction, *x*_u_, for PFO‐ (•) and P(F8:F1/4)‐dodecane (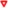) gels. For the sake of clarity, (a) reports *T* values only for the endotherm with the highest peak heat flow. The temperature at which neat PFO undergoes the nematic‐isotropic melt transition (○) is also indicated in (a) (top right corner); the corresponding transition is not seen for P(F8:F1/4) before the onset of thermal decomposition at 340 °C. Dashed lines in (b) represent a linear fit and a guide to the eye, respectively, for PFO and P(F8:F1/4) data.

Temperature‐dependent PL and light‐scattering measurements on solutions/gels were performed on a 90°‐optical geometry Jasco FP‐8500 spectrofluorometer equipped with a thermostated cuvette holder (ETC‐815). Polymer solutions in dodecane (*c*_p_ = 0.5 wt %) were sealed inside glass capillary tubes (Hilgenberg; 1.5 mm diameter) and mounted upright inside a 10 × 10 mm quartz cuvette (Hellma). The cuvette was then filled with hexadecane to provide thermal contact to the capillary. Confining the polymer solution within a capillary tube of small cross‐section was necessary to ensure (i) small optical density of the sample in order to reduce PL self‐absorption and (ii) minimal thermal inertia of the solution during homogeneous gelation after quenching below the dissolution temperature. Solutions were equilibrated for 15 min at 110 °C [above their dissolution temperature; see Fig. 3(a)]; PL spectra were recorded at this point to confirm that the solutions comprised well‐dissolved polymer chains with no observable emission due to β‐phase or crystalline chain segments. The solutions were then quenched to the crystallization temperature *T*_c_ = 50 °C, and the collection of PL and light‐scattering spectra (integration time ≈ 15 s) was initiated. For PL,  *λ*_ex_ = 440 and 433 nm were used for PFO and P(F8:F1/4) solutions, respectively, corresponding to spectral positions ∼3 nm higher than the peak absorption of β‐phase and crystalline chains as determined by UV‐Vis absorption measurements (cf. Fig. 2). For scattering, nonresonant excitation at *λ*_ex_ = 500 nm was used. Unpolarized excitation and detection were used for both measurements.

## RESULTS

### Solid‐State Thermal Properties

Figure 1 shows the chemical structures of PFO and P(F8:F1/4) and highlights the significant differences in solid‐state thermal properties associated with the selection of specific side‐chain substituents. Differential scanning calorimetry (DSC) thermograms for PFO are consistent with previous reports^21^, ^31^, ^32^ and show crystallization into two polymorphs (so‐called α‐ and α'‐phase) upon cooling from 300 °C (isotropic melt), with the nematic melt transition observed at ∼170 °C on subsequent heating. In contrast, P(F8:F1/4) does not crystallize upon cooling from 300 °C (nematic melt) but features a glass transition (*T*_g_ ≈ 100 °C), resulting in a “nematic glass” microstructure. The heating DSC trace of P(F8:F1/4) exhibits the features expected for a quenched, slowly‐crystallizing polymer such as poly(ethylene terephthalate) (PET).^58^ These include a glass transition at ∼100 °C and two broad crystallization peaks at 175 and 235 °C, followed by a nematic melt transition at ∼270 °C. The ∼100 °C difference in the nematic melting temperatures of PFO and P(F8:F1/4), as confirmed by temperature‐dependent optical microscopy (not shown here), may be attributed to lower melting entropy for P(F8:F1/4) due to its shorter side‐chains. The time‐of‐flight (TOF) hole mobilities measured for “glassy,” i.e. in‐plane isotropic, films of PFO and P(F8:F1/4) also differ by two orders of magnitude.^26^

### Dilute Solutions

Dilute solutions of both polymers in decalin, a moderately good solvent,^59^ were prepared with concentration (expressed as polymer weight fraction) *c*_p_ = 0.01 wt %, which is significantly below the calculated chain overlap concentration c* (see Experimental), thus minimizing the possibility of interchain interactions. Interestingly, the absorption and PL spectra of both solutions are identical (see Fig. 2) with a featureless absorption band centered at 391 nm and PL vibronic peaks at 415, 439, and 469 nm. The isolated chain conformation is expected to be wormlike for dilute solutions of both polymers in moderately good solvents,^9^, ^47^ resulting in comparable optical properties; the situation is very different for semidilute (*c*_p_ ≈ c*) solutions (*vide infra*). In addition, high (and equal) PL quantum efficiencies (PLQE ≈ 92 ± 1%) were measured for both solutions, confirming their chemical purity.

### Semidilute Solutions and Gels

Semidilute (*c*_p_ = 0.5 wt %) solutions of PFO and P(F8:F1/4) in decalin were stored at 5 °C over several days, yielding, respectively, a coherent yellow gel and a pale‐yellow suspension of microgels. We note, however, that, macroscopic gelation of P(F8:F1/4)‐decalin solutions was also possible at higher polymer concentrations. For the sake of simplicity, all solution‐crystallized polymer samples will hereafter be simply referred to as gels.

In comparison with that of the dilute solution, the PFO‐decalin gel absorption spectrum [cf. Fig. 2(a)] features two additional peaks at 437 and 405 nm, which indicate the presence of chain segments in the β‐phase conformation.^9^ Normalizing the gel and corresponding dilute solution absorption spectra at their short‐wavelength absorption tails (*λ* ≈ 300–330 nm) allows spectral subtraction to reveal the β‐phase chain segment absorption. The latter constitutes a relatively high fraction (∼40 %) of the spectrally‐integrated gel absorption, consistent with previous reports.^9^, ^60^, ^61^ The PL spectrum of the gel [cf. Fig. 2(c)] is wholly dominated by emission from the β‐phase segments and features a well‐defined vibronic progression with peaks at 439, 466 and 499 nm, fully consistent with previous reports for α‐pinene gels and dilute guest‐host dispersions in polystyrene^27^ and with there being efficient excitation state energy transfer to β‐phase segments.^37^ Compared with dilute solution, the PL spectrum of the β‐phase‐rich gel exhibits a reduced linewidth of the S_1_‐S_0_ 0‐0 vibronic, indicating a smaller degree of conformational disorder for the β‐phase relative to the wormlike conformation in solution.^30^, ^37^, ^62^ Together with the small Stokes shift between β‐phase absorption and PL 0‐0 vibronic peaks, this further suggests a rigid planar structure^30^, ^37^, ^62^ as is also consistent with the reduced Huang‐Rhys parameter (implying a small degree of geometric rearrangement between ground and excited states).^30^, ^37^, ^62^, ^63^ Finally, the observed red‐shift in emission peaks is consistent with an increased conjugation length.^30^, ^37^, ^62^

The absorption spectra of the P(F8:F1/4) gel and dilute solution, again normalized at their short‐wavelength absorption tails, are shown in Figure 2(b). The P(F8:F1/4) gel exhibits a peak at 396 nm, with a shoulder at ∼430 nm (confirmed by its negative numerical second derivative^34^, ^64^) that is attributed to absorption by solution‐crystallized chain segments.^27^, ^62^ There is no evidence for a β‐phase contribution. The PL spectrum of the gel, shown in Figure 2(c), is red‐shifted relative to the dilute solution due to preferential emission from the crystalline chain segments and features vibronic peaks at 433, 457, and 487 nm (consistent with reports for crystalline PFO films).^31^, ^62^ Again, there is no evidence for a β‐phase contribution. The corresponding Huang‐Rhys parameter and S_1_‐S_0_ 0‐0 vibronic linewidth are intermediate between those of the dilute solution and the β‐phase‐rich PFO gel, also consistent with previous comparisons for film samples.^31^, ^62^ For reference, we note that Chen et al.^31^ reported *melt‐crystallized* α'‐phase PFO films with an absorption shoulder at 426 nm and PL vibronic peaks at 432, 458, and 489 nm. This close correspondence strongly supports the deduction that solution‐crystallization of P(F8:F1/4) results in non‐solvated polymer crystals, with the role of the solvent limited to facilitating the chain mobility required for crystallization to occur.

Consistent with the absence of β‐phase chain segments for the P(F8:F1/4) samples, it is known that polyfluorenes disubstituted with 2‐ethylhexyl *branched* alkyl side‐chains also do not adopt the β‐phase conformation and exhibit substantially different behavior to PFO upon solution‐crystallization.^65^, ^66^ In addition, the statistical co‐polymer P(F8:F2/6) comprising a 1 : 1 ratio of fluorene units substituted with di‐*n*‐octyl (F8) and di(2‐ethylhexyl) (F2/6) side‐chains has also been reported not to form the β‐phase in semidilute MCH solutions.^66^

### Phase Behavior

Thermal analysis by DSC was used to further study the solution‐crystallization behavior of PFO and P(F8:F1/4) as a function of polymer fraction. For these and all subsequent measurements, polymer solutions were prepared in dodecane—a moderately bad solvent for PFO (Hildebrand solubility parameter *δ* = 7.8 cal^1/2^ cm^−3/2^)^67^ that has the additional advantage of a relatively high melting point (*T*_m_ = −10 °C), allowing solvent melting/dissolution experiments to be carried out.

Figure 3(a) shows dissolution temperatures, *T*, of PFO‐ and P(F8:F1/4)‐dodecane gels as a function of polymer concentration, expressed here as the polymer repeat unit molar fraction *x*_u_. To facilitate data comparison, $x_{u}^{\text{PFO}}$ is based on the molar mass of the F8 repeat unit, whereas $x_{u}^{P\left(F8:F1/4\right)}$ is calculated using *half* the molar mass of the copolymer repeat unit. As expected, *T* increases with *x*_u_ for both polymers.^68^ Unlike the situation for PFO, crystallization or melting of P(F8:F1/4) does *not* occur for *x*_u_ ≥ 0.5, indicating that solvent deficiency strongly hinders polymer crystallization due, most likely, to reduced chain mobility; this can also account for the absence of melt‐crystallization in P(F8:F1/4) [cf. Fig. 1(b)]. Representative DSC thermograms for PFO‐ and P(F8:F1/4)‐dodecane mixtures are shown in Supporting Information Figure S1. PL spectra recorded for semi‐dilute PFO‐ and P(F8:F1/4)‐dodecane solutions and gels are fully consistent with the results obtained for decalin‐based solutions and gels.

Solvent melting/dissolution experiments, in which slowly‐crystallized gels, i.e. those possessing maximal degrees of crystallinity (see Experimental), are further cooled below *T*_m_ of the solvent and then reheated, recording the melting enthalpy of the solvent, Δ*H*_m_, were described in *Part I* of this study. Briefly, Δ*H*_m_ quantifies the amount of “free,” i.e. crystallizable, solvent which, in accordance with Gibbs' phase rules, must decrease linearly with *x*_u_ in the case of solution‐crystallization by polymer‐solvent compound formation.^69^, ^70^ As shown in Figure 3(b), the variation of Δ*H*_m_ with *x*_u_ for PFO‐dodecane can be described by a single linear fit with Δ*H*_m_ = 0 at *x*_u_ ≈ 0.5, which indicates the formation of a polymer‐solvent compound with stoichiometry of 1 : 1, i.e. one dodecane molecule per one F8 repeat unit. On the other hand, the corresponding Δ*H*_m_ data for P(F8:F1/4)‐dodecane does not show linear variation with *x*_u_, indicating that solution‐crystallization proceeds by an alternative mechanism involving the formation of non‐solvated polymer crystals, such as those that can typically be obtained by crystallization from the melt. In this case, the solvent is dispersed in the amorphous polymer regions. Hence, at increasing polymer concentrations, a higher fraction of solvent molecules would be isolated on a molecular level within the amorphous polymer phase and thus unable to crystallize, leading to the observed non‐linear decrease in Δ*H*_m_ with *x*_u_.

### X‐Ray Diffraction

**Figure 4:**
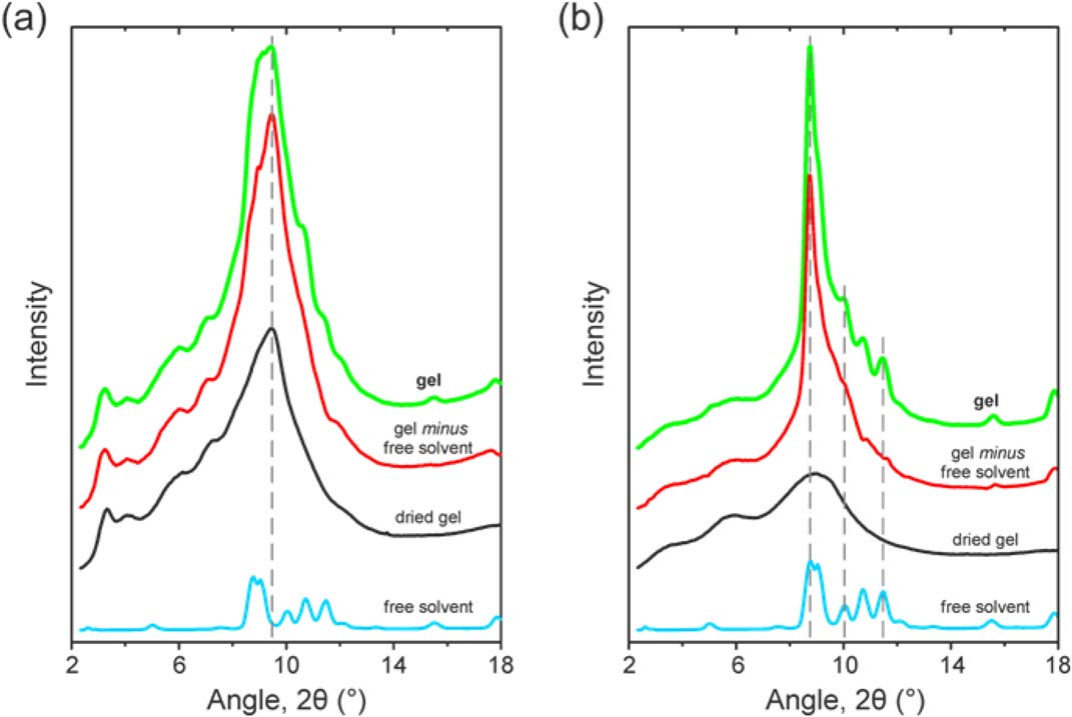
Radially‐integrated WAXD patterns recorded for (a) PFO (*x*_u_ = 0.48) and (b) P(F8:F1/4) (*x*_u_ = 0.33) gels in dodecane (green lines) at −100 °C (below *T*_m_ of the solvent and the expected *T*_g_ of the compound), together with the corresponding normalized diffraction patterns of the dried gels (black lines) and the free solvent (blue lines). Also shown are the diffraction patterns of the gels following subtraction of the normalized diffraction patterns of the free solvent (“gel *minus* free solvent”; red lines). The dashed vertical lines are a guide to the eye for the relative positions of the reflection peaks observed for the gels and the free solvent.

The solution‐crystallization mechanisms proposed above for the two polymers are corroborated by the results of a wide‐angle X‐ray diffraction (WAXD) analysis of slowly‐crystallized gels, presented in Figure 4. Diffraction patterns of the gels comprise contributions from two distinct components: (i) polymer‐solvent compound or non‐solvated polymer crystals, together with any residual amorphous polymer fraction and (ii) crystals of the “free,” i.e. non‐intercalated and/or crystallizable, solvent. In order to minimize the contribution from the latter, we subtracted the normalized diffraction patterns of the neat solvent from that of the as‐prepared gels (cf. “gel *minus* free solvent” data in Fig. 4). A detailed description of the WAXD analysis is given in *Part I* of this study.

For comparison, diffraction patterns were also recorded for dried polymer gels obtained by critical‐point drying of the as‐prepared gels. This technique allows for interface‐free removal of solvent and minimizes the possibility of associated structural changes to the polymer.^56^, ^57^

As was the case for other PFO gels (see *Part I*), the diffraction patterns of both the as‐prepared gels subtracted with the free‐solvent contribution and the dried gels, shown in Figure 4(a), feature a strong reflection at *d* = 0.42 nm that is *unique to the gels* and does *not* have a counterpart in the diffraction pattern of the free solvent. In *Part I* the peak at *d* = 0.42 nm was attributed, in part, to the (004) reflection associated with the *c*‐axis periodicity of the intercalated solvent within the compound.

On the other hand, although the diffraction pattern of the P(F8:F1/4)‐dodecane gel also features distinct reflections, these do not match those observed for the dried gel and instead are found to *closely correspond to the diffraction pattern of the free solvent*, as shown by the dashed lines in Figure 4(b), albeit with some changes to the relative intensities of the peaks. This indicates that the solvent in P(F8:F1/4)‐dodecane gel is *not* involved in a co‐crystallized compound phase and is instead dispersed in the amorphous polymer regions.

Thus, based on the results of optical spectroscopy, as well as thermal and WAXD analysis, we conclude that solution‐crystallization of PFO and P(F8:F1/4) results, respectively, in solvated (i.e. polymer‐solvent compound) and non‐solvated polymer crystals. The following sections will examine the influence of the two different crystal types on gel microstructure and crystallization kinetics.

### Microstructure

**Figure 5:**
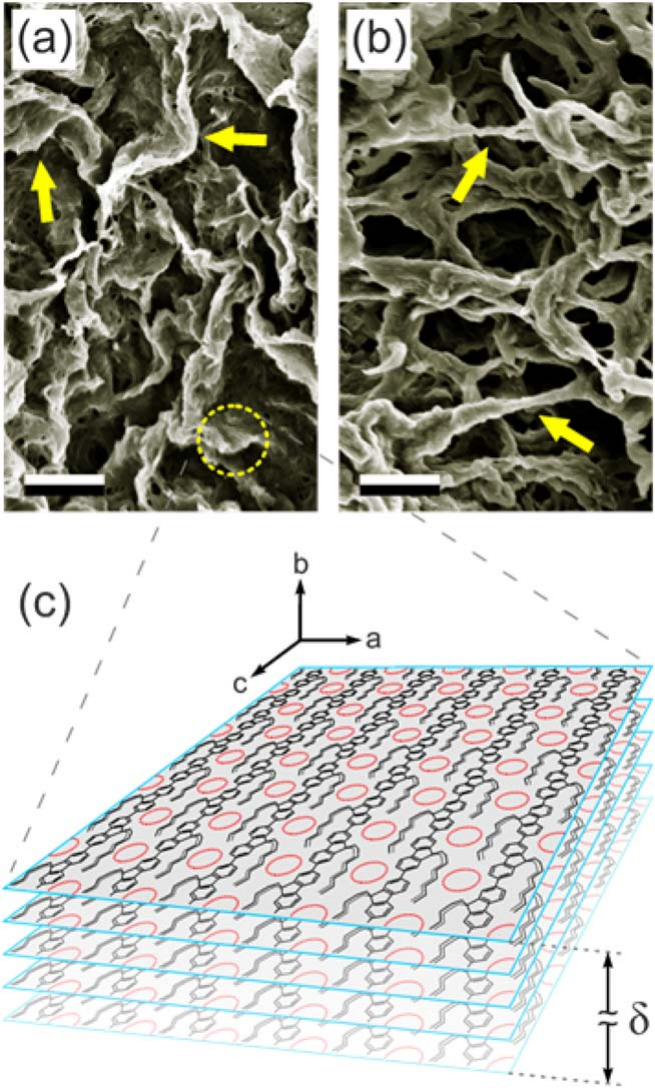
SEM images of aerogels obtained by critical‐point drying of polymer gels in decalin containing 0.5 wt % (a) PFO and (b) P(F8:F1/4). Arrows indicate the typical sheet‐ and fiber‐type features, respectively. Scale bars correspond to 1 μm. (c) Schematic illustration of the molecular arrangement proposed for the sheet‐like crystalline domains formed for the PFO–solvent compound: β‐phase chain segments and the intercalated solvent molecules are represented by black line chemical structures and red circles respectively, with *δ* indicating sheet thickness.

Aerogels of both polymers were fabricated by critical‐point drying of the gels prepared from semidilute (*c*_p_ = 0.5 wt %) decalin mixtures. Their microstructure, which is expected to be fully representative of polymer organization in the as‐prepared gels, was characterized by scanning electron microscopy (SEM). In agreement with the distinct spectroscopic and diffraction differences observed for the as‐prepared gels of the two polymers, the microstructures of their aerogels are also dissimilar, as shown by the SEM images in Figure 5. The PFO aerogel [cf. Fig. 5(a)] comprises sheet‐like structures (typical sheet thickness *δ* = 50–100 nm and lateral dimensions >1 μm), whereas a more fibrillar microstructure is observed for the P(F8:F1/4) aerogel [cf. Fig. 5(b); typical fiber diameter ϕ ≈ 100–200 nm]. Additional SEM images are presented in Figures S2 and S3 in the Supporting Information.

The formation of sheet‐like structures in solution‐crystallized PFO has previously been proposed on the basis of both small‐angle neutron scattering (SANS)^47^, ^61^, ^65^ and SEM^71^ measurements, with the sheets invariably displaying the optical and crystallographic characteristics of the β‐phase conformation.^47^, ^72^ Notably, Knaapila et al.^72^ reported that the fluorene backbones within the sheets are  π–π‐stacked perpendicular to the sheet plane, in the sheet thickness direction. Elsewhere, Liu et al.^43^, ^73^ provided a detailed analysis of orthorhombic solution‐grown β‐phase crystals of F8 oligomers. On the basis of these reports and the demonstration of PFO–solvent compound formation in *Part I* of this study, the molecular organization within solution‐crystallized PFO sheets is proposed to be as depicted in Figure 5(c). A single layer in the sheet comprises β‐phase chain segments extended along the *c*‐axis and assembled along the *a*‐axis, with the phenylene rings oriented in the *ac*‐plane and the solvent molecules intercalated into on‐chain cavities. Given that our preparation of PFO‐solvent compounds closely matched that of Liu et al.^43^ who reported that solution‐grown β‐phase crystals correspond to an orthorhombic crystalline structure irrespective of solvent choice and quality,^73^ we propose that the structure of PFO‐solvent compounds similarly corresponds to an orthorhombic unit cell. In this case, subsequent layers would be packed along the *b*‐axis with, most likely, π–π stacking of adjacent β‐phase fluorene backbones and a concomitant alignment of the intercalated solvent molecules. The resulting microstructure within the solution‐crystallized PFO sheets would then feature a molecular‐level co‐crystalline arrangement of the polymer and the small‐molecular (solvent) species. This predicted double‐oriented microstructure would also be highly anisotropic, with the polymer/solvent layers *alternating* along the *a*‐axis and *continuous* along the *b*‐axis. A more detailed X‐ray diffraction study will be needed, however, to fully determine the crystal structure of the sheet‐like domains in PFO–solvent compounds.

It is interesting to compare the microstructure of the PFO–solvent compound, as revealed by its aerogel, with the typical observations for other polymer‐solvent compounds. While a broad range of microstructures has been reported, fibrillar‐type compounds are most commonly observed.^74^ This aspect of microstructure has been extensively documented for sPS‐based compounds. For example, Daniel et al.^56^ studied sPS gels prepared from chloroform and 1‐chlorotetradecane (*c*_p_ = 10 wt % in each case), which feature, respectively, solvated (i.e. polymer‐solvent compound) and non‐solvated polymer crystals. Chloroform‐based aerogels displayed a fibrillar microstructure (fiber diameter = 50–100 nm), with similar observations reported for sPS compounds with other solvents such as tetrahydrofuran, 1,2‐dichloroethane, and trichloroethylene.^57^ On the other hand, non‐solvated polymer crystals in chlorotetradecane‐based sPS aerogels featured a sheet‐like microstructure (sheet thickness = 200–400 nm). Interestingly, the reverse situation is observed for polyfluorene aerogels in Figure 5. This is likely to be related to the differences in chemical structure and, consequently, the range of stable chain conformations for sPS and the polyfluorenes used in this study. The 2_1_‐helical geometry of the β‐phase chain conformation of PFO is inherently “ribbon‐like”, such that sheet‐like PFO‐solvent compound crystals are to be expected; conversely, fiber‐formation can be expected for other conformational motifs such as are present in solution‐crystallized P(F8:F1/4). The similarities to secondary structures in proteins are evident, although in that case hydrogen bonding between chain segments plays an important role.

### Crystallization Kinetics

Having demonstrated the differences in solution‐crystallization for the two polyfluorenes, we use time‐ and temperature‐dependent spectroscopy to investigate the kinetics of these processes. Semidilute solutions offer a trade‐off between chain mobility, facilitated due to a low density of entanglements, and adequate signal intensity.

**Figure 6:**
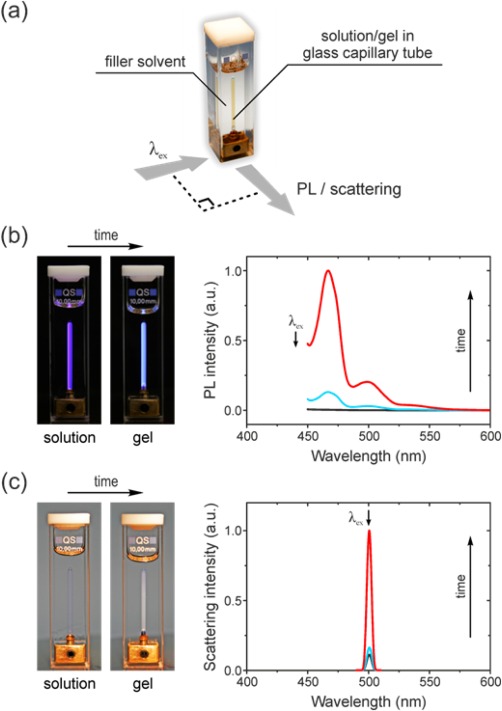
(a) Schematic illustration of the cuvette‐based sample holder and 90° measurement geometry used for time‐ and temperature‐dependent optical spectroscopy on 0.5 wt % polyfluorene solutions/gels in dodecane. Representative PL and light‐scattering data for a PFO solution quenched to 50 °C are shown in (b) and (c) respectively. The excitation wavelength, *λ*_ex_, is indicated in each case. PL and scattering spectra are reported for the solution (black line, smallest signal), and at the corresponding onset (blue line) and completion (red line, largest signal) of gelation. Photographs of the solution and the gel under (b) UV and (c) white‐light illumination are also shown.

**Figure 7:**
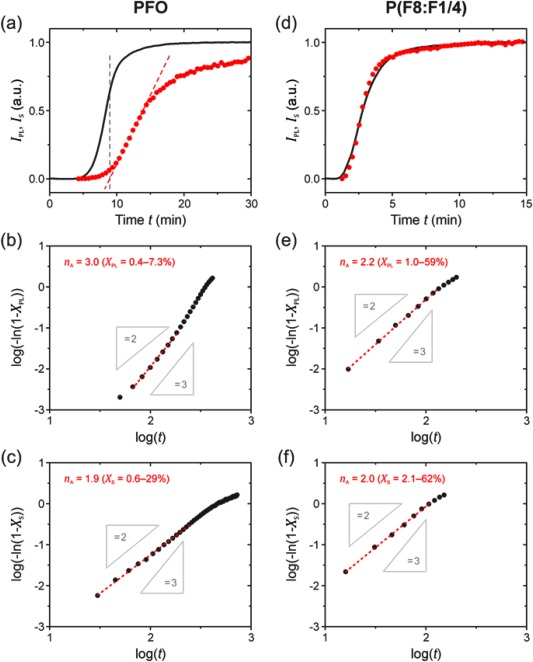
Time‐dependent PL and light‐scattering measurements performed on dodecane solutions containing 0.5 wt % PFO (left column) and P(F8:F1/4) (right column) after quenching from 110 to 50 °C at time *t* = 0, thereby initiating crystallization/polymer‐solvent compound formation. (a, d) Integrated PL (*I*_PL_, black lines) and light‐scattering (*I*_S_, red circles, baseline‐corrected) intensities as a function of *t*. Avrami plots are shown for the corresponding (b, e) PL and (c, f) light‐scattering data. Experimental data (•) is shown for the 0.1 ≤ *X* ≤ 80% range, where *X* is the relative degree of transformation. Linear fits to the data are also shown (dashed red lines), with the gradient *n*_A_ (Avrami exponent) value and the fitted *X* range indicated in each case.

To this end, 0.5 wt % solutions of both polymers in dodecane were sealed inside glass capillary tubes, thus minimizing their thermal inertia, and then mounted inside a cuvette filled with neat solvent in order to provide thermal contact. The cuvette was placed inside a commercial 90° excitation/detection geometry spectrofluorometer as illustrated in Figure 6(a), with the cuvette temperature controlled by the instrument's software. Quenching the solutions below their dissolution temperatures initiated crystallization/gelation, involving two distinct steps:

1. *Intra*‐chain structure formation, i.e. adoption by chain segments of a particular conformation that is found in the resulting crystal. As was shown in Figure 2, changes in the chain conformation are evidenced by distinct changes in absorption and PL emission spectra. In order to monitor conformational changes during crystallization/gelation, the quenched solutions were photoexcited directly at the absorption peak/shoulder corresponding to solution‐crystallized chain segments and the resulting PL intensity,* *was recorded at regular time intervals. Photoexcitation wavelengths were 440 and 433 nm for PFO and P(F8:F1/4) solutions respectively; their spectral position is indicated by the arrows in Figure 2(a,b). As shown in Figure 6(b) for a PFO solution quenched to 50 °C, the PL excited at *λ*_ex_ = 440 nm increases from essentially zero (solution) to a well‐defined spectrum (fully transformed gel) that features the characteristic β‐phase vibronic progression. Solutions of both polymers have negligible absorption at the respective *λ*_ex_ values [cf. Fig. 2(a,b)]. Thus, site‐selective excitation and the resulting absence of significant excitation energy transfer from amorphous/dissolved chains imply that the integrated PL intensity may be used to quantify the relative fraction of PFO β‐phase or P(F8:F1/4) crystalline chain segments that are formed during the solution‐crystallization process.
2. *Inter*‐chain structure formation, i.e. incorporation of chain segments into a crystalline lattice. As confirmed by the representative photographs in Figure 6(c), the solutions of both polymers are transparent outside the absorbing spectral region while the gels are turbid due to scattering by polymer‐solvent compound or polymer crystals. In order to monitor the emergence of crystals in quenched solutions, the scattering intensity for non‐resonant excitation (*λ*_ex_ = 500 nm) was recorded as a function of time. Representative data is shown in Figure 6(c) for PFO solutions quenched to 50 °C. The integrated intensity of the scattering peak increases by a factor of 9 as the solution transforms into a crystalline gel. Due to imperfect index matching, however, the scattering intensity for the solution [Fig. 6(c), black curve] is nonzero and this signal was taken as the baseline in our subsequent analysis (Fig. 7).

For further measurement details the reader is directed to the Experimental section as well as Figures S4–S6 in the Supporting Information. We note that similar 90° geometry spectrofluorometer measurements have been reported by others: For instance, Liao et al.^75^ studied the different stages of filament formation for tau protein in solution by monitoring time‐dependent fluorescence and scattering. Elsewhere, Saha et al.^76^ investigated the kinetics of riboflavin‐melamine supramolecular complex formation by using PL measurements to follow the solution‐gel transition. Finally, Huang et al.^77^ used similar time‐dependent fluorescence measurements to study the phase behavior of a low‐molecular‐mass organogelator in dodecane. In the two latter examples, Avrami analysis^78^, ^79^, ^80^ was successfully applied to extract detailed information about crystallization kinetics.

Figure 7(a) and (d) show the time‐evolution of the integrated PL, *I*_PL_, and scattering, *I*_S_, intensities for quenched PFO and P(F8:F1/4) solutions respectively. All curves feature a characteristic sigmoidal profile. Of particular note is that for PFO the increase in PL intensity occurs substantially *prior* to the increase in scattering. The onset of the latter approximately corresponds to the saturation in PL intensity, as indicated by the dashed lines in Figure 7(a). The scattering signal eventually reaches a plateau at *t* ≈ 90 min. These features indicate that *intra*‐chain order, i.e. the change in chain conformation, develops prior to *inter*‐chain crystallization, implying that the formation of β‐phase chain segments is *not* driven by *inter*‐chain interactions. On the other hand, the data for P(F8:F1/4) [cf. Fig. 7(d)] shows that, within experimental error, the PL and scattering intensity curves overlap, both reaching a plateau at *t* ≈ 15 min. One might thus infer that *intra*‐chain conformational ordering and *inter*‐chain crystallization are closely linked in the case of P(F8:F1/4).

Since the described experiments correspond to isothermal crystallization conditions, Avrami analysis can be applied to further highlight the differences in solution‐crystallization behavior of the two polymers. The data in Figure 7(a,d) was fitted using the well‐known Avrami equation:^78^, ^79^, ^80^

(3) $$\ln\left(1-X\left(t\right)\right)=-k_{A}t^{n_{A}}$$

where *X*(*t*) is the relative degree of *intra*‐ or *inter*‐chain transformation at time *t*, *k*_A_ is a rate constant, and *n*_A_ is the so‐called Avrami exponent corresponding to a particular nucleation type and growth geometry.

The Avrami plots for PFO are shown in Figure 7(b,c). The kinetics of β‐phase chain segment formation (i.e. *intra*‐chain ordering) in Figure 7(b) are well described by *n*_A_ = 3 which can correspond to either (i) sporadic nucleation and two‐dimensional growth or (ii) predetermined nucleation and three‐dimensional growth. We consider the former scenario to be less likely since the two‐dimensional (disk‐like) growth geometry appears to be illogical. Hence we infer that nucleation of β‐phase segments is predetermined, meaning that the nuclei develop simultaneously upon cooling to the crystallization temperature, followed by spherical (i.e. isotropically‐occurring) growth. On the contrary, *n*_A_ ≈ 2 is found for the *(inter*‐chain) PFO‐solvent compound crystallization [cf. Fig. 7(c)]. We interpret the latter, on the basis of the observed compound formation and resulting microstructure, as sporadic nucleation and predominantly one‐dimensional crystal growth, envisaged to be orthogonal to the chain‐extended β‐phase segment axis [cf. Fig. 5(c)]. The difference in the two Avrami exponents further confirms that β‐phase segment formation is independent of, and takes precedence over, subsequent inter‐chain assembly into polymer‐solvent compound crystalline domains.

As expected, in the case of P(F8:F1/4) identical Avrami exponents are observed for both *intra*‐chain conformational ordering and *inter*‐chain crystallization [cf. Fig. 7(e,f) respectively]. The value of *n*_A_ ≈ 2 corresponds to sporadic nucleation and predominantly one‐dimensional growth, envisaged to be orthogonal to the long axis of the chains.

We note that the observed time‐dependence of *intra*‐ and *inter*‐chain structure formation, as well as the corresponding *n*_A_ values, were also found for other crystallization/quenching temperatures (see additional data in Supporting Information Fig. S7). Furthermore, the Avrami exponents for *inter*‐chain crystallization of (more concentrated) solutions, as well as neat PFO, were confirmed to be *n*_A_ ≈ 2 by DSC analysis, the results of which are presented in Figures S8 and S9 in the Supporting Information.

**Figure 8:**
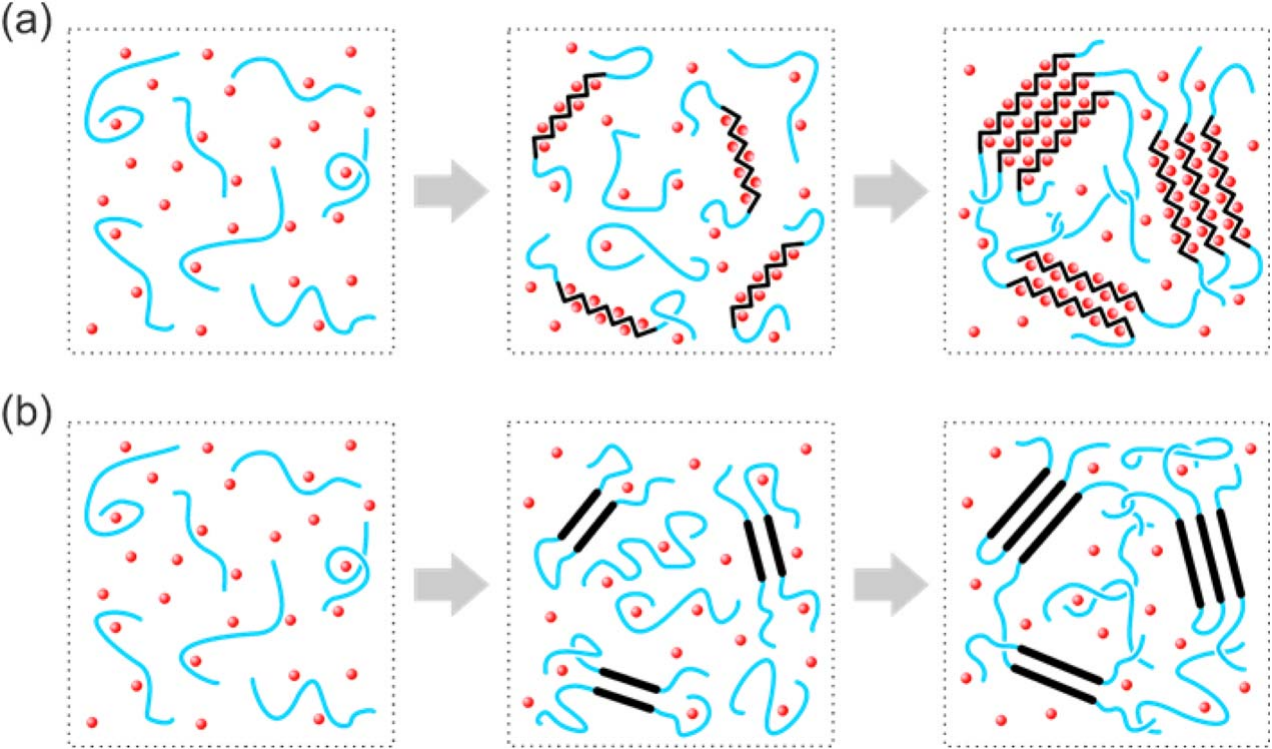
Schematic illustration of the steps occurring for solution‐crystallization of (a) PFO and (b) P(F8:F1/4). Disordered polymer chains and solvent molecules are depicted, respectively, as blue lines and red dots; polymer chain segments with intra‐chain order are depicted as black zigzags or lines, following Ref. 81. Left panels show the identical starting solutions; central panels show the formation of primary nuclei, and right panels show the final stage of crystal growth and the accompanying thermoreversible gelation.

To summarize the observations above, solution‐crystallization of both polyfluorenes is schematically illustrated in Figure 8. In the case of PFO, solution‐crystallization by polymer‐solvent compound formation involves two discrete steps [cf. Fig. 8(a)], both of which presumably minimize the system's Gibbs free energy. As the first step, upon cooling the solution to a certain temperature, a fraction of isolated wormlike PFO chains undergo *intra*‐chain ordering, forming β‐phase segments with the small‐molecular solvent simultaneously intercalated into on‐chain cavities. This physically‐bound solvent stabilizes the β‐phase conformation in the absence of *inter*‐chain interactions. Predetermined nucleation together with adoption of the specific chain geometry required for the formation of a stoichiometric polymer‐solvent compound imply that, instead of evolving gradually from a disordered wormlike state, the β‐phase conformation of PFO is intrinsically well‐defined and largely crystallization‐condition‐invariant in terms of its inter‐monomer torsion angle. The reduced entropy of a PFO solution rich with highly ordered, isolated β‐phase chain segments in turn increases the thermodynamic driving force for *inter*‐chain association and leads to the second solution‐crystallization step, namely the formation of solvated sheet‐like β‐phase crystals.

In the case of non‐solvated polymer crystals formed during solution‐crystallization of P(F8:F1/4), shown schematically in Figure 8(b), the chain conformation is stabilized only by its simultaneous incorporation into a crystalline lattice. Therefore, the primary nuclei are most likely to be pairs of chain segments, formed either as a result of chain folding or *inter*‐chain association, that feature both *intra*‐ and *inter*‐chain conformational/crystalline order. Subsequent crystal growth proceeds by attachment of additional conformationally‐ordered chain segments to the primary nuclei. For both polyfluorenes, crystal growth continues until the mobility of residual disordered chains is arrested by macroscopic thermoreversible cross‐linking (i.e. gelation) of the solutions, subject to polymer concentration and degree of undercooling.

## DISCUSSION

There are several implications of the results presented in this study that deserve additional comment, pertaining both to our understanding of the β‐phase conformation of PFO and to our ability to realize controllable solution‐processing of polyfluorenes.

First, our findings corroborate the reports by Grell et al.^27^ and Da Como et al.^51^ of β‐phase formation in isolated PFO chains dispersed in an inert polymer matrix upon exposure of the samples to solvent vapor. In the latter study the β‐phase conformation was found to preferentially form in already‐extended chain segments. This is consistent with PFO‐solvent compound formation; the intercalation of solvent should be facilitated by the minimal need for structural rearrangement. The fact that the formation of β‐phase occurs independently of *inter*‐chain interactions also has an important implication for the recently‐demonstrated sub‐micron‐scale spatial patterning of the β‐phase conformation in PFO films by localized exposure to liquid solvent.^59^ Our results suggest that the ultimate resolution limit of that dip‐pen nanolithography‐based patterning technique may be the physical dimensions of a single PFO chain.

Second, the reported details of PFO solution‐crystallization inform new approaches to growing large crystals. Such samples would feature maximal fractions of highly‐ordered β‐phase chain segments with concomitant attractive optoelectronic properties. As demonstrated recently for poly(3‐hexylthiophene) (P3HT),^82^ the fabrication procedure is likely to be based on ultraslow crystallization of PFO from dilute solutions at low undercooling, leading to correspondingly low nucleation densities. Alternatively, the use of a crystallizable solvent and crystallization under thermal gradients can also be advantageous in allowing directional epitaxial solution‐crystallization.^83^ Cross‐linkable linear side‐chains could, provided solvent‐polymer compound formation is not disturbed, enable the crystalline ordered β‐phase structure to be locked‐in.^36^

## CONCLUSIONS

In summary, we have demonstrated fundamental differences in solution‐crystallization behavior for two polyfluorenes resulting from changes to the structure of their alkyl side‐chains.

Solution‐crystallization of PFO, substituted with *linear* octyl side‐chains, occurs via polymer‐solvent compound formation and involves two discrete steps, as demonstrated by *in‐situ* spectroscopic monitoring of isothermal crystallization. Our results show that the first step involves the formation of isolated β‐phase chain segments in the absence of significant inter‐chain aggregation/crystallization due to the chain conformation being stabilized by physically‐bound solvent intercalated into on‐chain cavities. This step is characterized by an Avrami exponent *n*_A_ = 3, corresponding to predetermined nucleation and three‐dimensional growth. The reduction in entropy due to the presence of comparatively‐rigid β‐phase chain segments leads to the second crystallization step, namely inter‐chain aggregation/crystallization of β‐phase segments, which is described by an Avrami exponent *n*_A_ = 2, corresponding to sporadic nucleation and one‐dimensional growth.

In the case of P(F8:F1/4), introducing *branched* 2‐methylbutyl alkyl side‐chains on alternating fluorene repeat units prevents the formation of the β‐phase conformation and results in an alternative conformational motif, the spectroscopic signature of which bears similarity to that of PFO crystallized from the nematic melt. The aforementioned chain conformation does not allow for the formation of on‐chain cavities and, therefore, solution‐crystallization of P(F8:F1/4) is found to result in non‐solvated polymer crystals. In this crystallization process, both intra‐ and inter‐chain order (that is, chain conformation and polymer crystals, respectively) develop simultaneously since, in the absence of co‐crystallization with the solvent, both of these structural elements are required for the formation of stable crystal nuclei. For isothermal solution‐crystallization conditions, both intra‐ and inter‐chain crystallization of P(F8:F1/4) are described by *n*_A_ = 2, corresponding to sporadic nucleation and one‐dimensional growth.

Our findings clarify the nature of the β‐phase chain conformation of PFO, corroborating that it is distinct from other conformational isomers. In solution, the solvent molecules can physically bind at predetermined positions (cavities) along the β‐phase segment, thereby stabilizing the precisely‐defined chain geometry.

Looking ahead, it would be of interest to use a broader range of scattering techniques to further study the interplay between intra‐ and inter‐chain structure formation during solution‐crystallization of PFO. Investigating how a judicious control of solution‐crystallization conditions can be used to control the microstructure of β‐phase domains should also be beneficial for optimizing the optoelectronic properties of solution‐processed PFO‐based devices.

## Supporting information

Supplementary Information
