# Supplementary material for: Solution‐crystallization and related phenomena in 9,9‐dialkyl‐fluorene polymers. II. Influence of side‐chain structure
Source: J Polym Sci B Polym Phys. 2015 Aug 17;53(21):1492–506. doi: 10.1002/polb.23797 (PMC4975719; doi:10.1002/polb.23797)
Supplement: Supplementary file 1 — Supplementary Information [file POLB-53-1492-s001.pdf]

Supporting Information

"Solution-Crystallization and Related Phenomena in 9,9-Dialkyl-Fluorene Polymers. II. Influence of Side-Chain Structure"

Aleksandr Perevedentsev e*t al.*

1. DSC Measurements of PFO and P(F8:F1/4) Mixtures in Dodecane.

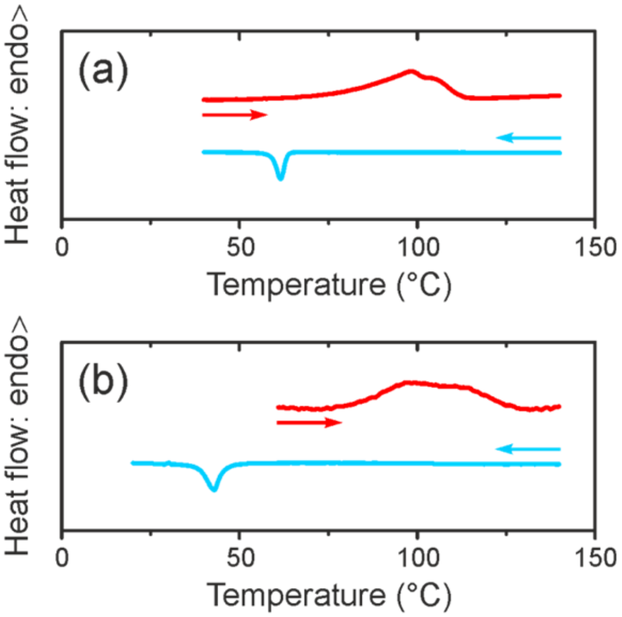

FIGURE S1 Representative first-cooling (blue lines) and second-heating (red lines) DSC thermograms recorded for **(a)** 35 wt % PFO and **(b)** 30 wt % P(F8:F1/4) mixtures in dodecane. Peak heatflow magnitudes are normalized for clarity.

Crystallization is observed as a single exotherm in the cooling thermograms. Dissolution/melting is observed as two overlapping endotherms in the heating thermograms; these are due to the occurrence of dynamic polymer recrystallization/melting prior to complete dissolution (see *Part I* of this study for further details).

2. Scanning Electron Microscopy of PFO and P(F8:F1/4) Aerogels.

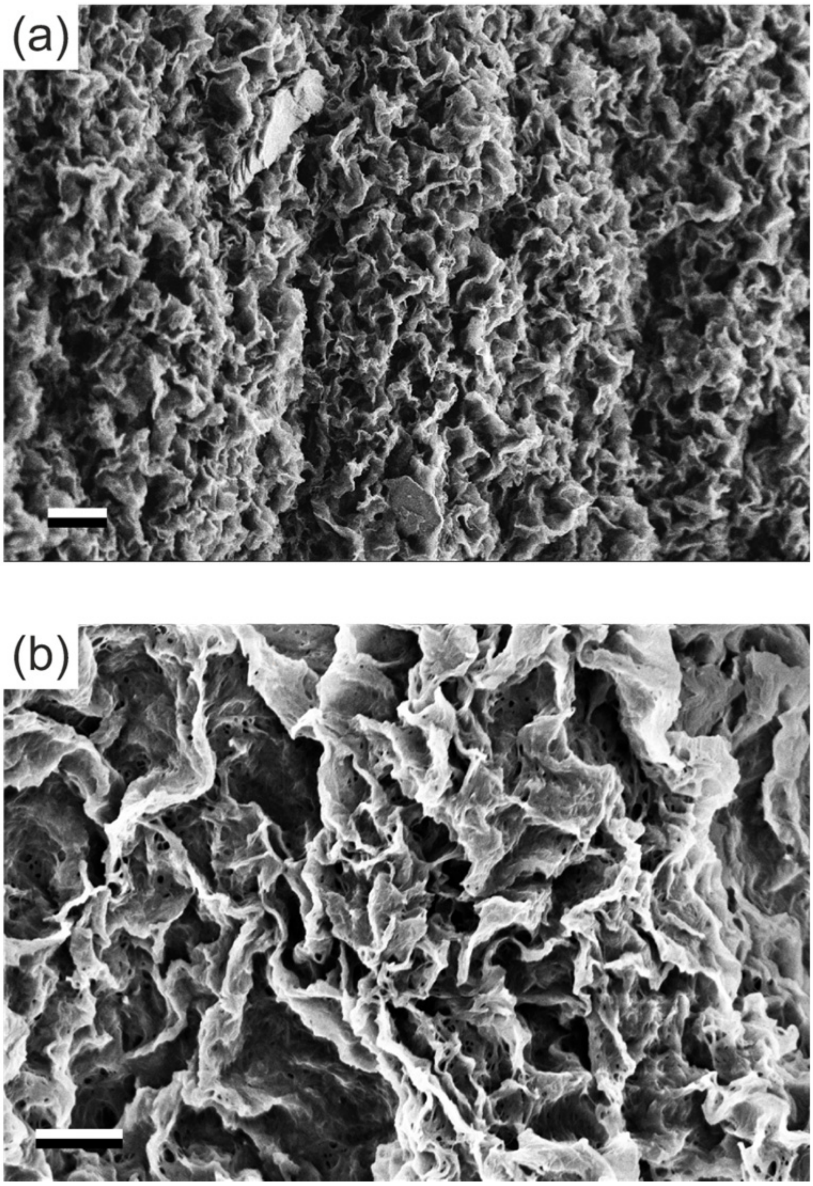

FIGURE S2 SEM images of the aerogel obtained by critical-point drying of 0.5 wt % PFO gel in decalin. The scale bars in **(a)** and **(b)** correspond to 2 μm and 1 μm respectively. Sheet-like polymer structures are evident.

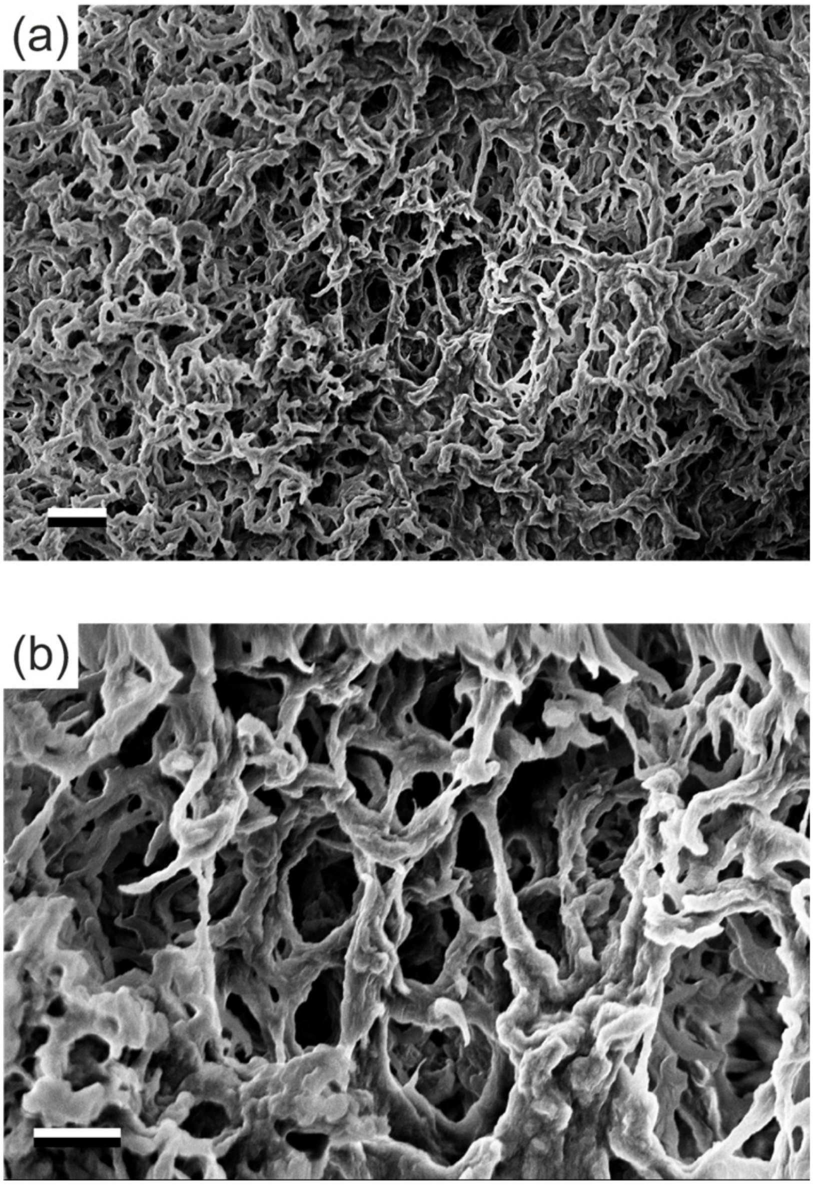

FIGURE S3 SEM images of the aerogel obtained by critical-point drying of 0.5 wt % P(F8:F1/4) gel in decalin. The scale bars in **(a)** and **(b)** correspond to 2 μm and 1 μm respectively. Fibrillar polymer structures are evident.

3. Time- and Temperature-Dependent PL and Light-Scattering Measurements in the 90°-angle Spectrofluorometer.

Figure S4 shows the PL spectra of 0.5 wt % solutions and gels of PFO and P(F8:F1/4) in dodecane. As for the (more dilute) 0.01 wt % solutions in decalin (see Figure 2(c) in the main text), the PL spectra of both solutions are identical, indicating that both polymers adopt similar disordered wormlike conformations above the dissolution temperature. The relative magnitude of the S_1-S_0 0-0 vibronic peaks is reduced due to self-absorption. The S_1-S_0 0-1 vibronic peaks of both solutions are essentially unaffected by self-absorption due to minimal spectral overlap (absorption edge of solutions is at ~425 nm; cf. Figure 2(a)) and are centered at 435 nm, which is ~4 nm higher than for the corresponding 0-1 peaks recorded for 0.01 wt % solutions in decalin. This discrepancy is minor and is likely to be caused by concentration- or solvent-related effects.

PL spectra recorded for the gels are identical to the corresponding PL spectra of the gels in decalin shown in Figure 2(c), albeit with stronger self-absorption of the S_1-S_0 0-0 vibronic peaks due to higher optical densities of the samples used for these measurements. For PFO and P(F8:F1/4) gels in dodecane, the S_1-S_0 0- 1 vibronic peaks appears at 466 and 458 nm, respectively.

Finally, we note that neither solutions nor gels show any appreciable green-band (so-called "g-band") emission, indicating that thermo-oxidative degradation of the polymers is likely to be negligible.

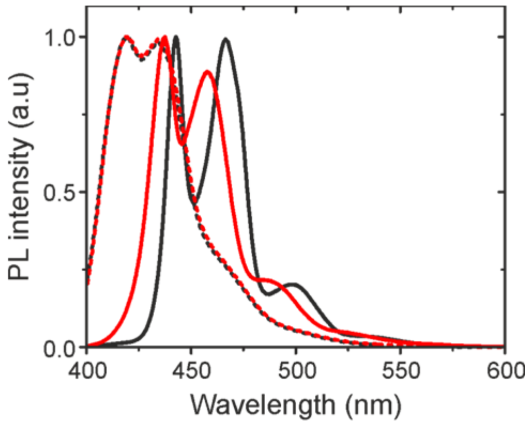

FIGURE S4 Peak-normalized PL spectra (λ_{ex} = 390 nm) for 0.5 wt % solutions/gels of PFO (black lines) and P(F8:F1/4) (red lines) in dodecane. Data is shown for isotropic solutions at 110 °C (dotted lines) and fully-transformed gels at 40 °C (solid lines), for which the temporal evolution of PL and scattering intensities has saturated.

Light-scattering spectra of solutions and gels *outside the absorbing spectral region* were measured as the so-called "synchronous" spectra, in which excitation and emission wavelengths in the 90°-angle fluorometer are scanned simultaneously, with the scattered intensity I_S recorded as a function of wavelength. Representative data for solutions and fully-transformed gels is presented in Figure S5(a,b) for PFO- and P(F8:F1/4)-based mixtures respectively. While it is beyond the scope of this study to elucidate the physical origin of the observed peaks (e.g. at ~500 and 540 nm), it is immediately clear that I_S is consistently higher for gels compared with isotropic solutions. Taking the ratio of I_S recorded for the respective gels and solutions yields the light-scattering ratio spectra shown in Figure S5(c). The I_S ratio spectra for both PFO- and P(F8:F1/4)-based mixtures feature a similar shape, albeit with a slight offset, indicating that the I_S contrast between gel and solution is somewhat higher for P(F8:F1/4). For both polymers, the ratio of I_S recorded for gels and solutions increases for reduced λ_{ex}.

Therefore, λ_{ex} = 500 nm was selected as the optimal excitation wavelength for time-dependent light-scattering measurements, since it satisfies the criteria of (i) being outside the absorbing spectral region and (ii) featuring an increased I_S contrast between gels and solutions, thereby allowing for higher signal-to-noise ratios.

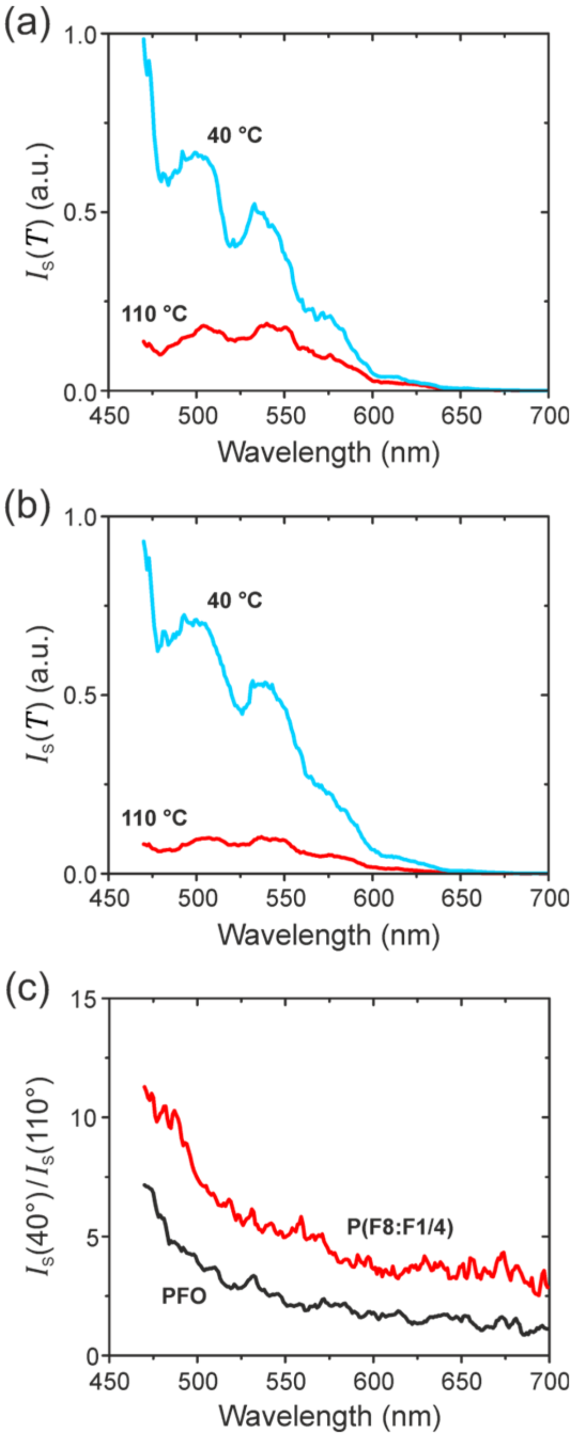

FIGURE S5 Light-scattering spectra, measured by synchronous scanning of excitation and emission wavelengths in the spectrofluorometer (excitation and emission slit width = 2.5 nm; relative wavelength offset = 0 nm), for 0.5 wt % solutions/gels of **(a)** PFO and **(b)** P(F8:F1/4) in dodecane, showing the scattered intensity I_S at the indicated temperatures T. Measurements at 110 °C and 40 °C correspond to solutions and equilibrated gels, respectively. The ratio of I_S spectra measured at 40 and 110 °C are shown in **(c)** for both polymers.

In order to confirm that time- evolution of I_S during solution-crystallization is essentially independent of λ_{ex}, we have recorded I_S for a 0.5 wt % PFO–dodecane solution quenched to 50 °C at three different λ_{ex}: 500, 600 and 700 nm. The results are presented in Figure S6 and show identical time-evolution of I_S for the different non-resonant excitation wavelengths.

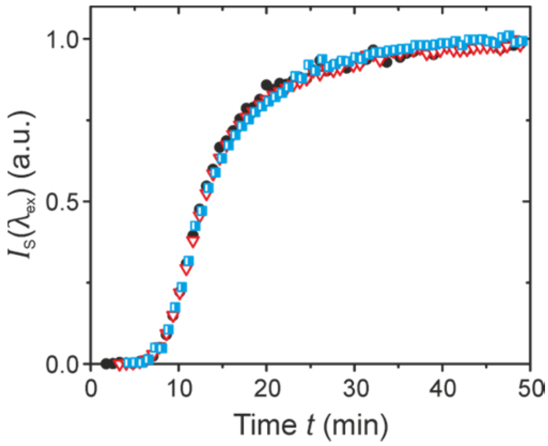

FIGURE S6 Integrated light-scattering intensities $I_S(\lambda_{ex})$ recorded for 0.5 wt % PFO–dodecane solutions after quenching to 50 °C at time t = 0; data is shown for excitation wavelengths λ_{ex} = 500 (●), 600 (▽) and 700 nm (◨). Identical time-evolution of I_S is observed for all three (non-resonant) λ_{ex}.

Figure 7 in the main text presented the time-evolution of PL and scattering intensities for PFO and P(F8:F1/4) solutions in dodecane that were quenched to 50 °C, thus inducing polymer crystallization. The data obtained by these isothermal crystallization measurements was analyzed using the Avrami equation with the aim of extracting the Avrami exponent n_A, which provides information on the corresponding nucleation type and growth geometry. Figure S7 provides a counterpart to Figure 7 in the text and presents complementary data collected at lower crystallization temperatures. In order to follow the faster crystallization kinetics at higher undercooling, it was necessary to reduce the data acquisition frequency (and, therefore, spectral integration times) to ~8–10 s, leading to somewhat higher experimental error margins. Nevertheless, the data in Figure S7 fully corroborates both the relative differences in time-evolution of intra- and inter-chain structure formation during crystallization and the corresponding n_A values.

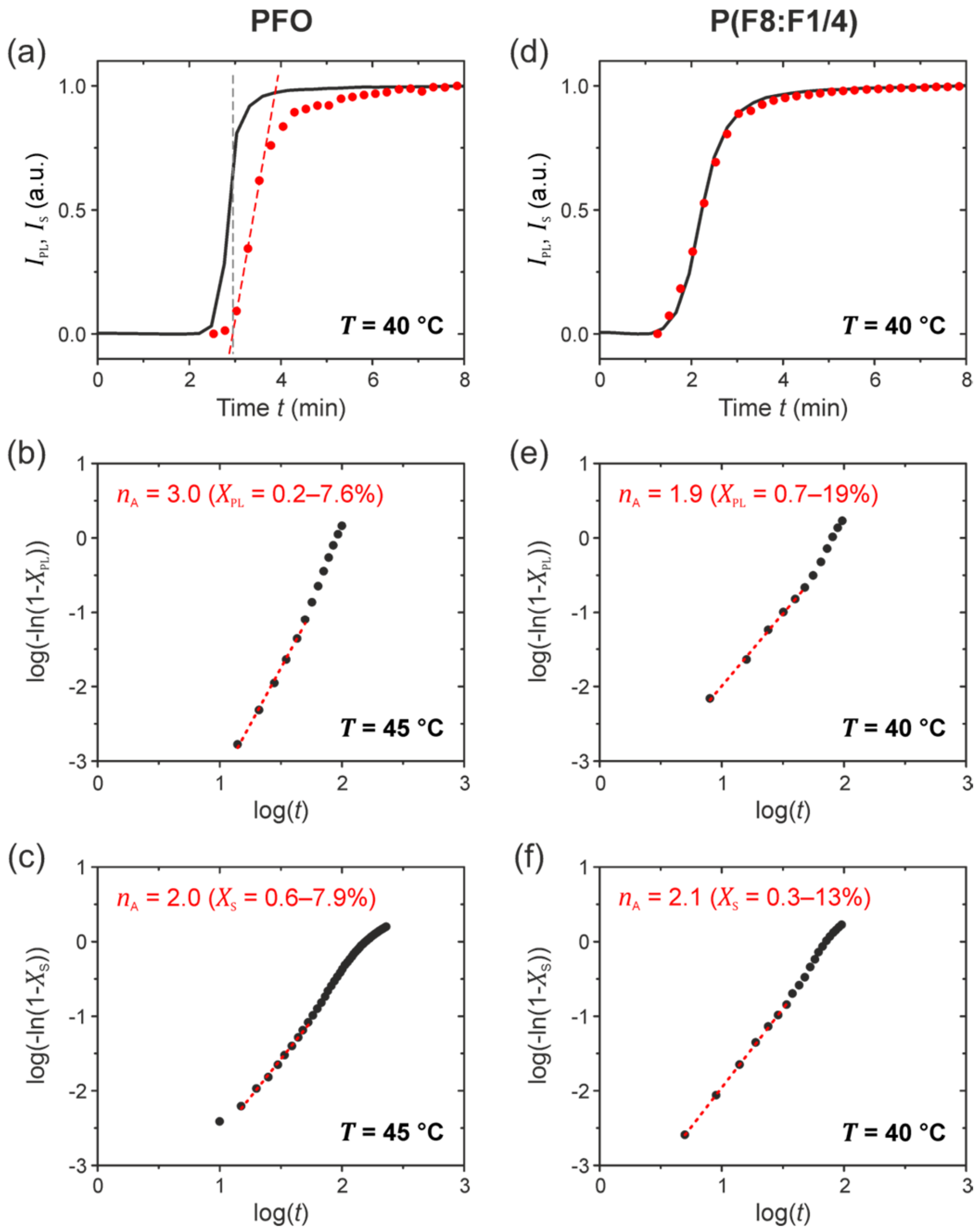

FIGURE S7 Time-dependent PL and light-scattering measurements performed on 0.5 wt % solutions of PFO **(left column)** and P(F8:F1/4) **(right column)** in dodecane after quenching to temperature T (T < dissolution temperature) at time t = 0, inducing crystallization. The quench temperatures T are indicated in each case. **(a, d)** Integrated PL (I_{PL}, black lines) and light-scattering (I_S, red circles) intensities as a function of t. Avrami plots for the corresponding **(b, e)** PL and **(c, f)** light-scattering data. Experimental data (●) is shown for the $0.1 \leq X \leq 80\%$ range, where X is the relative degree of transformation. Linear fits to the data (dotted red lines) are also shown, with gradient n_A, corresponding to the Avrami exponent, and the fitted X range indicated in each case.

4. Avrami Analysis of Isothermal Crystallization by DSC Measurements.

In order to confirm the Avrami exponents for *inter*-chain crystallization, determined by monitoring the time-evolution of scattering intensities for quenched solutions, additional isothermal crystallization experiments were carried out using DSC. In order to obtain a sufficiently high heatflow signal in the DSC, it was necessary to use more concentrated solutions, that is c_p = 11 and 30 wt % for PFO and P(F8:F1/4) solutions in dodecane, respectively. The results presented in Fig. S8 confirm that $n_A \approx 2$ for *inter*-chain crystallization of both PFO- and P(F8:F1/4)-based mixtures.

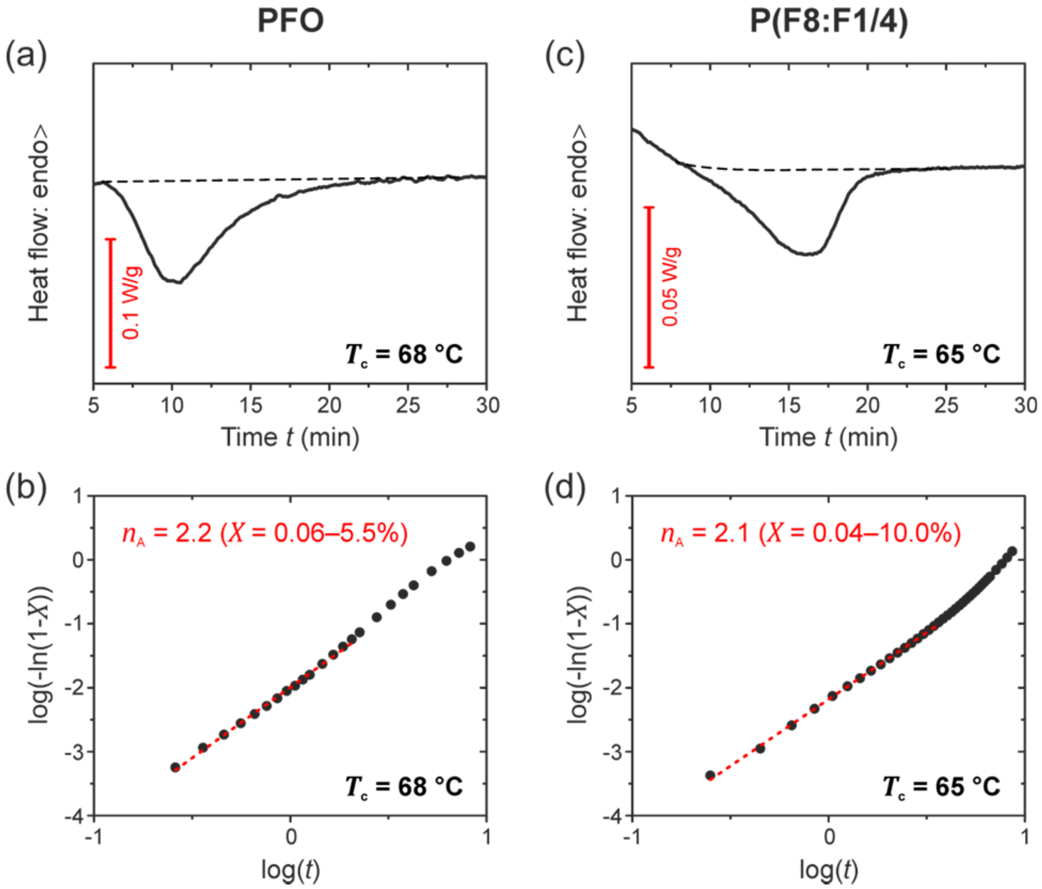

FIGURE S8 Isothermal crystallization experiments and Avrami plots for 11 wt % PFO **(left column)** and 30 wt % P(F8:F1/4) **(right column)** solutions in dodecane. The solutions were quenched to their respective crystallization temperatures T (indicated in each case) at $t = 0$. **(a, c)** Crystallization exotherms recorded by DSC and the corresponding spline-type baselines are shown by solid and dashed lines respectively. Heat flow scale bars are also shown in each case. **(b, d)** Avrami plots for the corresponding DSC data. Experimental data (●) and linear fits (dotted red lines) are shown, with gradient n_A, corresponding to the Avrami exponent, and the fitted X range indicated in each case. X represents the degree of transformation, quantified by the relative crystallinity in the sample at time t.

For completeness, Fig. S9 also shows the DSC thermogram of isothermal melt-crystallization of neat PFO and the corresponding Avrami plot. The analysis yielded $n_A \approx 2$ which corresponds to sporadic nucleation and one-dimensional growth.

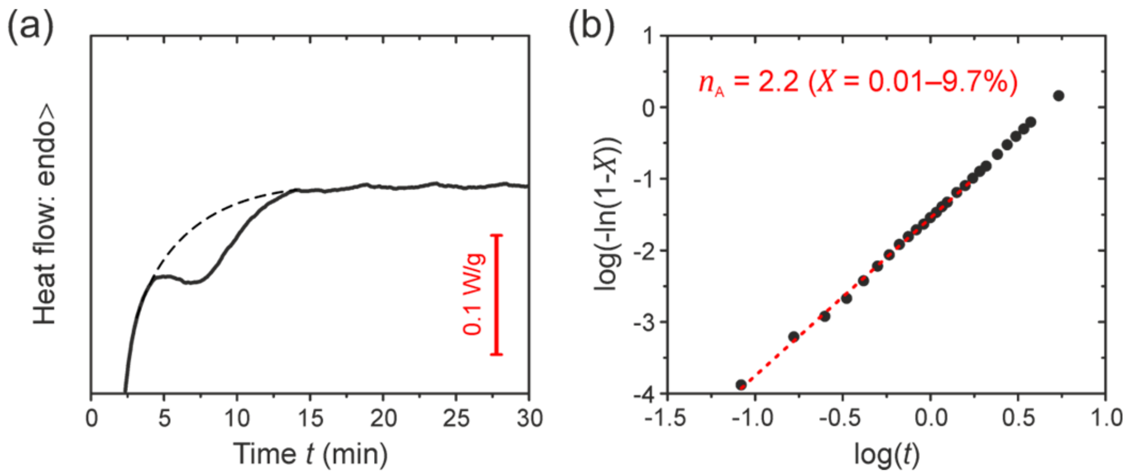

FIGURE S9 Isothermal crystallization experiments and Avrami plots for neat PFO, carried out at T = 152 °C following quenching at t = 0 from the nematic melt at 200 °C. **(a)** DSC thermogram (solid line) and the corresponding spline-type baseline (dashed line). **(b)** Avrami plot, showing experimental data (●) and the linear fit (dotted red line) in the indicated X range; gradient n_A, corresponding to the Avrami exponent, as also given.
